# Dietary acidifiers blend modulates growth, antioxidant, immunity, cytokine performances, and the disease control in Nile tilapia fingerlings fed on high-soybean feeds

**DOI:** 10.1007/s10695-025-01598-5

**Published:** 2025-11-11

**Authors:** Mohsen Abdel-Tawwab, Riad H. Khalil, Rania Nasr, Adel H. Saad, Nehad M. S. Mahmoud, Nashwa Abdel-Razek

**Affiliations:** 1 Department of Fish Biology and Ecology, Central Laboratory for Aquaculture Research, Agricultural Research Center, Abbassa, Abo-Hammad, Sharqia, 44662 Egypt; 2 Department of Poultry and Fish Diseases, Faculty of Veterinary Medicine, Alexandria University, Alexandria, Egypt; 3 Department of Fish Health and Management, Central Laboratory for Aquaculture Research, Agricultural Research Center, Abbassa, Abo-Hammad, 44662 Sharqia Egypt; 4 Department of Nutrition and Clinical Nutrition, Faculty of Veterinary Medicine, Matrouh University, Matrouh, Egypt; 5 Department of Aquaculture, Faculty of Fish and Fisheries Technology, Aswan University, Aswan, Egypt

**Keywords:** Dietary acidifiers, Nile tilapia, Growth enhancement, Antioxidant and immune function, Bacterial infection

## Abstract

The present research examined the use of a commercial acidifier blend (CAB) as a feed supplement on growth, digestive enzymes, antioxidant, immunological biomarkers, and defense of Nile tilapia (*Oreochromis niloticus*) against *Aeromonas sobria* disease. Fish (22.7 ± 0.76 g) were fed on 0.0 (the control), 1.0, 2.0, 4.0, or 8.0 g CAB/kg feed for 60 days. After that, fish were intraperitoneally injected with *A. sobria*, and their survival was monitored for a further 10 days. The fish group that received 4.0 g CAB/kg feed showed the highest growth rate. The improved growth in this group is correlated with the upregulation of mRNA expressions of growth-related genes (*IGF-1* and *GH*). In addition, the highest activities of intestinal proteases, lipase, and α-amylase were observed in fish fed with 4.0 g CAB/kg feed. Compared to the control group, fish fed on CAB-containing diets showed significantly higher activities of superoxide dismutase (SOD), catalase (CAT), glutathione peroxidase (GPx). In a parallel way, the mRNA expression of *SOD*, *CAT*, and* GPx* genes were upregulated in CAB-fed groups, particularly at levels of 4.0–8.0 g CAB/kg feed. Conversely, malondialdehyde levels were maximized in the control group but were reduced in CAB-fed fish. Additionally, dietary CAB, especially at levels of 4–8 g/kg diet progressively enhanced the immune function and upregulated the expression of *IL-1ß*, *IL-8*, and* IL-10* genes. The bactericidal and challenge tests against *A. sobria* evoked that CAB exhibited antibacterial properties; the maximum mortality rate following the bacterial infection was noted in the control group (90%); meanwhile, CAB-fed fish, especially at a rate of 4.0–8.0 g/kg feed exhibited lower mortality rates (40% and 45%, respectively). Overall, this study recommends incorporating 4.0 g CAB/kg feed to mitigate the negative impacts of dietary soybean and improve the growth, antioxidant, and immunological indices in Nile tilapia fingerlings.

## Introduction

Nile tilapia (*Oreochromis niloticus*) is one of the most farmed freshwater fish species in Egypt, from which it was distributed into many countries worldwide because of its suitability for sustainable aquaculture (El-Sayed 2020; Emam et al. 2025). As tilapia farming expands, there is increasing pressure to reduce its production costs, particularly feed costs, to make the industry more sustainable and profitable. Recently, there has been a great interest in using plant-derived components including soybean meal (SBM) as protein sources in fish diets instead of fishmeal (FM). Animal diets often include SBM as a plant-derived protein resource, accounting for > 70% of global plant-based meal manufacturing, totalling about 236 million metric tons annually (AAS 2020). It is quite low cost compared to FM, making it an attractive option for aquafeeds production, but SBM contains antinutritional factors that can retard fish growth and impair their health. The inclusion of SBM in fish diets with high levels can trigger inflammatory responses, disrupting intestinal balance, and altering gut microbiota structure in various fish species (Trushenski 2015; Ringø et al. 2016; Zhu et al. 2021). Therefore, developing nutritional strategies to enhance intestinal health in SBM-fed fish is crucial. Generally, a single acidifier is used in aquafeeds to mitigate the detrimental impacts of high SBM levels in fish diets, while this study evaluated the use of an acidifier blend (formic acid and probiotic acid/salt) in aquafeeds and its impacts on the performance, innate immunity, and gene expression in Nile tilapia fingerlings.

*Aeromonas* spp. are rod-shaped, gram-negative, and motile microorganisms that can cause severe infections and mass mortality in fish (Yu et al. 2010; Abdel-Latif et al. 2020). Among them, *A. sobria* causes hemorrhagic septicemia in freshwater fish, including tilapia (Kozińska 2007; Bakr et al. 2021). Antibiotic and chemotherapeutic substances are usually used to control pathogens but they have been banned recently because of their deteriorating influences on fish health (Salma et al. 2022; Elgendy et al. 2024). Hence, the search for eco-friendly alternatives is necessary for managing and controlling infectious agents that negatively impact the aquaculture industry (Bondad-Reantaso et al. 2023; Elgendy et al. 2024).

Formic acid (FA)/salts have antimicrobial and growth-promoting properties, contributing to their benefits in Nile tilapia (Chen et al. 2024; Kassab et al. 2025), Asian sea bass, *Lates calcarifer* (Reyshari et al. 2019), rohu, *Labeo rohita* fingerlings (Sidiq et al. 2023), juvenile beluga *Huso huso* (Sayah et al. 2024), and gilthead sea bream, *Sparus aurata* (Torrecillas et al. 2024). Moreover, propionic acid (PA)/salts have demonstrated the ability to augment growth performance, boost innate immunity, and boost defenses in the face of pathogens in Nile tilapia (El-Adawy et al. 2018), Caspian white fish (*Rutilus frisii kutum*) fry (Hoseinifar et al. 2016), European seabass (*Dicentrarchus labrax*) fry (Wassef et al. 2020), and beluga sturgeon, *Huso huso* (Ahmadifar et al. 2022). The inclusion of acidifiers including FA t/salts or PA/salts lowers the gut pH, triggers the secretion of digestive enzymes, and modulates beneficial gut microbiota while reducing pathogenic ones (Hoseinifar et al. 2017; Tran et al. 2020; da Silva et al. 2023; Torrecillas et al. 2024; Hoseini et al. 2025). It is hypothesized that combining FA + PA/salt acidifiers in this study would synergistically affect tilapia growth and innate immunity and promote its resistance to *A. sobria* challenge. Therefore, this study aimed to evaluate the effects of a commercial acidifier blend (CAB) containing FA + PA/salt on growth performance, digestive enzymes, antioxidant status, immune responses, and resistance to *A. sobria* in Nile tilapia fed with high-SBM diets.

## Materials and methods

### Diets formulation and fish handling procedures

The CAB product was procured from Acidbac™ (Dex ibèrica, Vila-Seca, Tarragona, Spain), containing formic acid (150 g/kg) and propionic acid + calcium propionate (30 g/kg). It was supplemented to isonitrogenous (30% crude protein; CP) and isolipidic (6.2%) diets at concentrations of 0.0 (the control), 1.0, 2.0, 4.0, and 8.0 g/kg feed (Table 1). The feed ingredients were well-mixed for 30 min with sufficient water amount and processed into a feed paste via a meat mincer. The resulting feed filaments were dehydrated and crinkled into 1–2-mm-diameter pellets at room temperature to be suitable for fish consumption regarding their size and mouth opening. Then, it is stored at − 4 °C pending usage. The proximate chemical composition of the experimental diets was done according the methods of AOAC (2005) and represented in Table 1.

**Table 1:** Ingredients and proximate analysis (%; on dry matter basis) of diets containing different levels of a commercial acidifier blend (CAB)

	CAB levels (g/kg feed)				
	0.0 (Control)	1.0	2.0	4.0	8.0
Ingredients					
Fish meal (72% CP)	80	80	80	80	80
Soybean meal (45.3%)	370	370	370	370	370
Wheat bran	100	100	100	100	100
Ground corn	190	190	190	190	190
Corn gluten	150	150	150	150	150
Corn oil	15	15	15	15	15
Fish oil	20	20	20	20	20
Vitamin premix^a^	15	15	15	15	15
Mineral premix^b^	15	15	15	15	15
Calcium diphosphate	10	10	10	10	10
Starch	20	20	20	20	20
Carboxymethyl cellulose	15	14.0	13.0	11.0	7.0
CAB	0	1.0	2.0	4.0	8.0
Total	1000	1000	1000	1000	1000
Chemical composition (g/kg)					
Moisture	9.15	9.17	9.13	9.15	9.17
Crude protein	31.88	30.86	30.84	30.88	31.86
Total lipids	6.18	6.38	6.52	6.18	6.38
Total ash	4.13	4.18	4.13	4.183	4.28
Crude fiber	3.58	3.58	3.58	3.58	3.58
Nitrogen free extract^c^	48.35	48.36	48.36	48.37	48.39
GE (MJ/kg)^d^	16.69	16.72	16.76	16.68	16.70

^a^Vitamin premix (per kg of premix): thiamine, 2.5 g; riboflavin, 2.5 g; pyridoxine, 2.0 g; inositol, 100.0 g; biotin, 0.3 g; pantothenic acid, 100.0 g; folic acid, 0.75 g; paraaminobenzoic acid, 2.5 g; choline, 200.0 g; nicotinic acid, 10.0 g; cyanocobalamine, 0.005 g; a-tocopherol acetate, 20.1 g; menadione, 2.0 g; retinol palmitate, 100,000 IU; cholecalciferol, 500,000 IU

^b^Mineral premix (g/kg of premix): CaHPO_4_.2H_2_O, 727.2; MgCO_4_.7H_2_O, 127.5; KCl 50.0; NaCl, 60.0; FeC_6_H_5_O_7_.3H_2_O, 25.0; ZnCO_3_, 5.5; MnCl_2_.4H_2_O, 2.5; Cu(OAc)_2_·H_2_O, 0.785; CoCl_3_.6H_2_O, 0.477; CaIO_3_.6H_2_O, 0.295; CrCl_3_.6H_2_O, 0.128; AlCl_3_.6H_2_O, 0.54; Na_2_SeO_3_, 0.03

^c^Nitrogen-free extract (calculated by difference) = 100 − (protein% + lipid% + ash% + fiber%)

^d^Gross energy (GE) was calculated from NRC (2011) as 16.7 kJ/g, 37.4 kJ/g, and 16.7 kJ/g for protein, lipid, and carbohydrates, respectively

Nile tilapia, *O. niloticus*, fingerlings (21.5–23.7 g) were obtained from the nursery ponds at the Central Laboratory for Aquaculture Research (CLAR) in Abbassa, Abo-Hammad, Sharqia, Egypt, and acclimatized to the wet lab conditions for 14 days while being given a control diet (30% CP). Following their acclimation to the laboratory environment, fish (22.7 ± 0.76 g) were randomly distributed into fifteen 100-L glass aquaria (15 fish/aquarium) to represent five treatments in triplicates. Aquaria were filled with dechlorinated tap water and supported with continual aeration via air pumps and air stones. Fish received the experimental diets thrice daily at 9:00, 13:00, and 17:00 h until they were apparently satiated for 60 days. The remaining feeds in each aquarium were siphoned 45 min post-feeding, dehydrated, weighed, and used to calculate the actual feed intake. A half of the aquarium’s water was siphoned daily to remove any remaining fish waste and exchanged with new freshwater from a storage reservoir. Water temperature, dissolved oxygen, pH, and unionized ammonia were all checked daily to ensure water purity. Water parameters were reserved within suitable ranges for Nile tilapia: dissolved oxygen (5.5–6.5 mg/L), pH (7.7–7.9), temperature (26.7–28.3 °C), and unionized ammonia (0.11–0.15 mg/L), consistent with recommended standards (Boyd and Tucker 2012).

Fish were fasted for 24 h after the feeding experiment and then anaesthetized with MS-222 (100 mg/L). The fish per each aquarium were collected, counted, and weighed in groups. Then, growth performance and feed utilization indices were calculated as follows:

- Weight gain (WG%) = 100 × (final weight − initial weight)
- Specific growth rate (SGR [%/day]) = 100 (Ln final weight − Ln initial weight)/60
- Feed conversion ratio (FCR) = total feed intake/weight gain
- Survival rate (%) = 100 × (final fish number/initial fish number)

### Blood and tissues sampling

Fish were fasted for 24 h at the end of the feeding trial and then anaesthetized as previously mentioned. Five fish were collected randomly from each aquarium (*n* = 15/treatment), and blood samples were collected from the caudal vessels and then aliquoted into two sets. The first set was treated with sodium heparin (500 U) for hematological examination, and the other set was centrifuged (5000 × g, 10 min at room temperature) to get serum, which was reserved at − 20 °C for further biochemical assays. Following blood sampling, the anaesthetized fish were humanely euthanized, rinsed, and dissected. Then, mid-intestinal and hepatic tissues were gathered and preserved at − 20 °C to evaluate intestinal digestive enzymes and hepatic antioxidative indices. Other liver and anterior kidney parts were carefully kept in 1.5-mL Eppendorf tubes having 500 μL of TRIzol®reagent (Invitrogen #15,596,026) and then stored at − 80 °C to assess genes expressions.

### Digestive enzymes activities

Mid-intestine samples were homogenized in saline solution (1 g: 9 mL), centrifuged, and the supernatants kept at − 20 °C. According to Bernfeld (1955), α-amylase values were evaluated through a solution of 1% starch in a Tris–HCl buffer (0.1 M, pH 7.0) as the standard solution. Following Kunitz (1947), the proteases level was assessed using a casein buffer substrate, and the supernatant absorbance was detected at *λ* = 280 nm. Hydrolysis of triglycerides in olive oil was used as a substrate to evaluate the lipase level (Cherry and Crandall 1932). According to Bradford (1976), the total protein (TP) values in the crude enzymes extract were measured, and the digestive enzymes’ activity was quantified as units per milligram of protein.

### Hemato-biochemical assays

The blood analysis was done following Dacie and Lewis’s methods (Dacie and Lewis 1984). White blood corpuscles (WBCs) and red blood corpuscles (RBCs) were monitored manually using a Neubauer hemocytometer under 400 × magnification with an optical microscope. Hemoglobin (Hb) concentration was evaluated using a diagnostic kit and spectrophotometry. Hematocrit (Ht) was calculated via centrifugation of the heparinized blood samples (1400 × g, 5 min). Mean corpuscular volume (MCV), mean corpuscular hemoglobin (MCH), and mean corpuscular hemoglobin concentration (MCHC) values were computed according to Dacie and Lewis (1984).

As stated by Bradford (1976) and Doumas et al. (1971), serum levels of total protein (TP) and albumin (ALB) were determined, respectively. Globulin (GLOB) levels were then calculated by subtracting ALB values from TP values. The method of Trinder (1969) was used to measure blood glucose, while the methods of Reitman and Franke (1957) were utilized for determining the values of aspartate aminotransferase (AST) and alanine aminotransferase (ALT) with an automated Siemens ADVIA 2400 biochemical analyzer.

### Antioxidant and lipid peroxidation assays

The fish liver was first rinsed multiple times in a cold NaCl (0.85%) solution to remove any impurities. About 100 mg wet liver tissue was homogenized per 1.0 mL of phosphate buffer (pH 7.4) using a tissue homogenizer at cold temperatures (0–4 °C). The resultant liver homogenate was centrifuged at 5000 × g for 20 min, and the supernatant was subsequently utilized for analyzing antioxidant parameters. Specifically, superoxide dismutase (SOD) values were evaluated based on the method of McCord and Fridovich (1969). Catalase (CAT) values were determined by monitoring the reaction of H_2_O_2_ at *λ* = 240 nm, as outlined by Aebi (1984). Glutathione peroxidase (GPx) values were analyzed according to Paglia and Valentine (1967) through the reaction of methyl catechol at *λ* = 340 nm. The content of lipid peroxidation parameter indicated by malondialdehyde (MDA) was measured based on themethod of Ohkawa et al. (1979) via the reaction of thiobarbituric acid at *λ* = 532 nm.

### Immune function assays

The lysozyme (LYZ) level was measured utilizing *Micrococcus luteus* as a substrate, where one unit of LYZ level was demarcated as the quantity of enzyme that performed a reduction in absorbance of 0.001 OD per minute in 1 mL of serum as outlined by Ellis (1990). Total immunoglobulin (total Ig) values were measured following the protocol outlined by Siwicki and Anderson (1993). Additionally, respiratory burst activity (RBA) was determined using the nitro-blue-tetrazolium (NBT) assay, which determines the production of intracellular oxidative free radicals, as described by Anderson and Siwicki (1995). Samples of heparinized blood (100 µL) were combined with 100 µL of 0.2% NBT (Sigma, St. Louis, MO, USA), and the resulting solution was thoroughly mixed and incubated for 30 min at 25 °C. Following the incubation and a second homogenization, 50 µL of the solution was transferred to 1 mL of N, N-dimethyl formamide (DMF, Sigma, St. Louis, MO, USA) in a glass tube. This new mixture was then homogenized and centrifuged at 3000 × g for 5 min. The optical density of the supernatant was measured using a spectrophotometer (Fischer, USA) at a wavelength of *λ* = 540 nm. The blank sample included the same components and procedures, but the blood was replaced with distilled water.

### Genes expressions

The tissues samples were carefully kept in 1.5-mL Eppendorf tubes having 500 μL of TRIzol®reagent (Invitrogen #15,596,026), and the total RNA extract was analyzed according to the manufacturer’s instruction. The mRNA expression levels of *IGF-I*, *GH*, *SOD*, *CAT*, *GPx*, *IL-1β*, *IL-8*, and *IL-10* were analyzed using real-time PCR. The RNA samples were treated with DNase I (Promega) to eliminate any contamination from genomic DNA. The purity of the isolated total RNA was assessed using UV spectrophotometry, with O.D. 260:280 ratios ranging from 1.8 to 2.0. Additionally, the purity of the total RNA was confirmed through 1% agarose gel electrophoresis utilizing 0.5 μg/mL ethidium bromide. The RNA was reverse-transcribed into complementary DNA (cDNA) using the SuperScript® cDNA synthesis kit (Life Technologies), adhering to the manufacturer’s instructions. The expression levels of selected genes, along with the housekeeping β-actin gene, were evaluated via RT-PCR (CFX96™ RT-PCR, Bio-Rad Laboratories, Inc.) in accordance with the manufacturer’s protocols. A total volume of 20 μL for the reaction mixtures was prepared, consisting of 10 μL of SYBR Green supermix, 5 μL of cDNA template, and 5 μL of primers at a concentration of 0.4 mM each, for RT-PCR conducted under the following conditions: 10 min at 95 °C, followed by 40 cycles of 15 s at 95 °C, 60 s at 60 °C, and concluding with a final run of 15 s at 95 °C, 60 s at 60 °C, and 15 s at 95 °C. The accuracy of all amplicons was verified through melt curve analysis post-amplification. The housekeeping β-actin gene served as a standard for normalizing cDNA sample loading. Transcription folds were normalized against the reference gene *β-actin* to ensure precise quantification using the 2^−ΔΔCT^ method (Schmittgen and Livak 2008). The primer sequences and conditions are detailed in Table 2.

**Table 2:** The primers’ design used for genes expression in the present study

Genes	Primer	Efficiency (%)	Amplified size (bp)	Accession number
*β-actin*	F:5′- AGCAAGCAGGAGTACGATGAG-3′	95	143	KJ126772.1
	R:5′- TGTGTGGTGTGTGGTTGTTTTG-3′			
*IGF-1*	F:5′- CACCCTCTCACTACTGCTGT-3′	95	233	EU272149.1
	R:5′- CACAGTACATCTCAAGGCGC-3′			
*GH*	F:5′- CTGGTTGAGTCCTGGGAGTT-3′	95	177	KT387598.1
	R:5′- CAGGTGGTTAGTCGCATTGG-3′			
*IL-8*	F:5′- GCACTGCCGCTGCATTAAG-3′	96	85	NM_001279704.1
	R:5′- GCAGTGGGAGTTGGGAAGAA-3′			
*IL-1β*	F:5′- CAAGGATGACGACAAGCCAACC-3′	92	149	XM_003460625.2
	R:5′- AGCGGACAGACATGAGAGTGC-3′			
*IL-10*	F:5′- CTGCTAGATCAGTCCGTCGAA-3′	98	94	XM_003441366.2
	R:5′- GCAGAACCGTGTCCAGGTAA-3′			
*SOD*	F: 5′- GGTGCCCTGGAGCCCTA-3′ R: 5′- ATGCGAAGTCTTCCACTGTC-3′	95	377	JF801727.1
*CAT*	F: 5′- TCCTGAATGAGGAGGAGCGA-3′ R: 5′- ATCTTAGATGAGGCGGTGATG-3′	96	232	JF801726.1
*GPx*	F: 5′- CCAAGAGAACTGCAAGAGA-3′ R: 5′- CAGGACACGTCATTCCTACAC-3′	98	180	FF280316.1

### The serum bactericidal activity

Following 2 months of the feeding experiment, the serum bactericidal effect against *A. sobria* was assessed using the Miles-Misra technique (Okada et al. 1999). The *A. sobria* isolate, sourced from the Poultry and Fish Diseases Department, Faculty of Veterinary Medicine, Alexandria University, Egypt, was cultivated in tryptone soy broth at 30 °C for 24 h. The bacteria were centrifuged, rinsed, and resuspended in gelatin veronal buffer. The bacterial density was set to 1 × 10^5^ cells/mL, and viability was confirmed by inoculating sequential dilutions on tryptone soy agar (TSA). Serum specimens were incubated with the bacterial suspension for 60 min at 30 °C, along with the control group (positive control without serum and negative control without bacteria). After incubation, the mixtures were diluted and plated on TSA for viability counts, which were assessed after 24 h.

### The bacterial challenge test

A pathogenic *A. sobria* strain, was isolated from dead Nile tilapia, recognized through the VITEK 2 system (BioMérieux, France), and cultivated at the Faculty of Veterinary Medicine, Alexandria University, Egypt. The LD50 of *A. sobria* (the lethal dose of the bacterium that kills 50% of infected fish) was determined and it was 1.0 × 10^5^ cells/mL. Next to the 60-day feeding experiment, fish from each treatment were collected and reallocated into 100-L aquaria at a density of 10 fish/aquarium (two replicates for each treatment). Fish were subjected to an intraperitoneal injection (IP) with 0.1 mL of the bacterial suspension (1.5 × 10^8^ cells/mL) as explained by Reda et al. (2016). As a negative control group, 5 fish were IP with saline solution instead of the bacterial suspension. After the IP injection, the fish were closely monitored for a further 10 days to track the onset of clinical signs, post-mortem lesions, and mortality rates. To ensure the cause of mortality, the pathogen was re-isolated from mortal fish, ensuring that the observed effects were due to the *A. sobria* infection. The protection conferred by *A. sobria* was assessed as described by Amend (1981).

### Statistical analyses

Data analyses involved testing for normality and variance homogeneity via Kolmogorov–Smirnov and Bartlett’s assessments, respectively. Then, data were subjected to one-way ANOVA to assess the impacts of dietary CAB on Nile tilapia fingerlings. Tukey’s HSD test was used to compare among treatments means at *P* < 0.05. The polynomial regression analysis was used to determine the optimal CAB level (Yossa and Verdegem 2015). For all analyses, SPSS version 26 (SPSS, Richmond, VA, USA) was utilized based on the procedures outlined in Dytham (2011).

## Results

### Growth indices and digestive enzymes activity

**Fig. 1:**
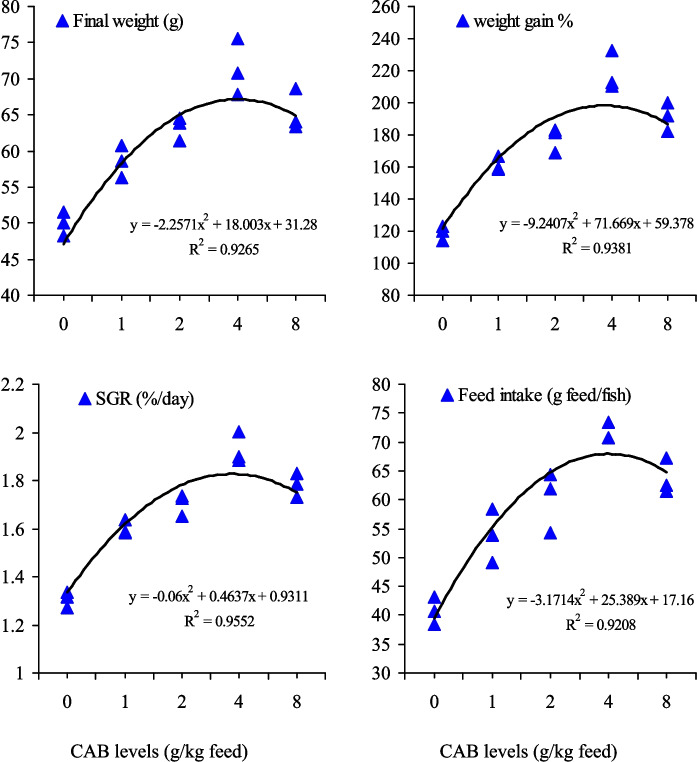
The relationship between final fish weight (g), weight gain %, specific growth rate (SGR; %/day), and feed intake of Nile tilapia (*O. niloticus*) fed on a commercial acidifiers blend (CAB) for 60 days

**Fig. 2:**
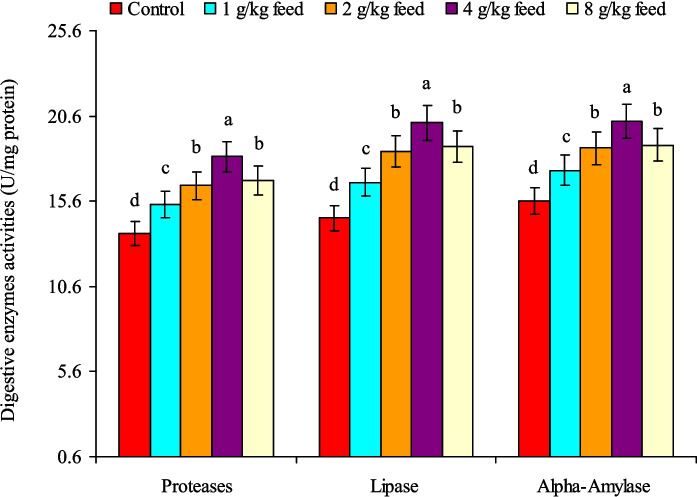
Changes in mid-intestinal digestive enzymes activities (U/mg protein) of Nile tilapia (*O. niloticus*) fed on a commercial acidifiers blend (CAB) for 60 days. Bars of each enzyme having different letters are significantly different at *P* < 0.05

The current investigation exhibited that fish provided with CAB-supplemented diets revealed a markedly (*P* < 0.05) higher growth performance than the control treatment (Table 3). Feeding the fish on 4.0 g CAB/kg feed resulted in highest final body weight, WG%, and SGR (%/day). Furthermore, fish fed on CAB-enriched diets demonstrated higher feed intake, especially at a level of 4 g CAB/kg feed with no impacts on FCR values, which remained between 1.48 and 1.50 (Table 3). No significant changes (*P* > 0.05) were detected in the fish survival rate (97.8%–100%). The polynomial regression fitting curve (Fig. 1) was used for final weight, WG%, SGR (%/day), and feed intake (g feed/fish), and it evoked that the optimum CAB level for Nile tilapia fingerlings is 4.0 g/kg feed. Additionally, CAB-containing diets enhanced digestive enzymes production (proteases, lipase, and α-amylase), and their highest activities were noticed in fish fed on 4.0 g CAB/kg feed, after which their activities declined as those fed on 2.0 g/kg feed (Fig. 2). The minimum activities of the abovementioned enzymes were noted in the control fish group (Fig. 2).

**Table 3:** Growth performance and somatic indices of Nile tilapia (*O. niloticus*) fed on different levels of a commercial acidifiers blend (CAB) for 60 days

Dietary CAB levels (g/kg feed)	Initial weight (g)	Final weight (g)	Weight gain %	SGR (%/day)	Feed intake (g feed/fish)	FCR	Fish survival (%)
0.0 (control)	22.7 ± 0.76	49.9 ± 0.95 d	119.8 ± 2.58 d	1.31 ± 0.020 d	40.7 ± 1.33 d	1.50 ± 0.086	97.8 ± 2.22
1.0	22.4 ± 0.31	58.6 ± 1.30 c	161.6 ± 2.65 c	1.60 ± 0.017 c	53.7 ± 2.65 c	1.48 ± 0.102	100.0 ± 0.00
2.0	22.8 ± 0.55	63.3 ± 0.96 bc	177.6 ± 4.46 b	1.70 ± 0.027 b	60.1 ± 3.04 bc	1.48 ± 0.065	100.0 ± 0.00
4.0	22.4 ± 0.25	71.4 ± 2.22 a	218.8 ± 7.10 a	1.93 ± 0.036 a	73.5 ± 1.62 a	1.50 ± 0.084	97.8 ± 2.22
8.0	22.4 ± 0.37	65.3 ± 1.68 b	191.5 ± 5.09 b	1.78 ± 0.029 b	63.8 ± 1.78 b	1.49 ± 0.081	100.0 ± 0.00
*P* value							
Linear	0.537	0.010	0.007	0.006	0.010	0.970	0.622
Quadratic	0.803	< 0.001	< 0.001	< 0.001	< 0.001	0.998	0.878

Mean values followed by different letters in the same column are significantly different at *P* < 0.05

**Fig. 3:**
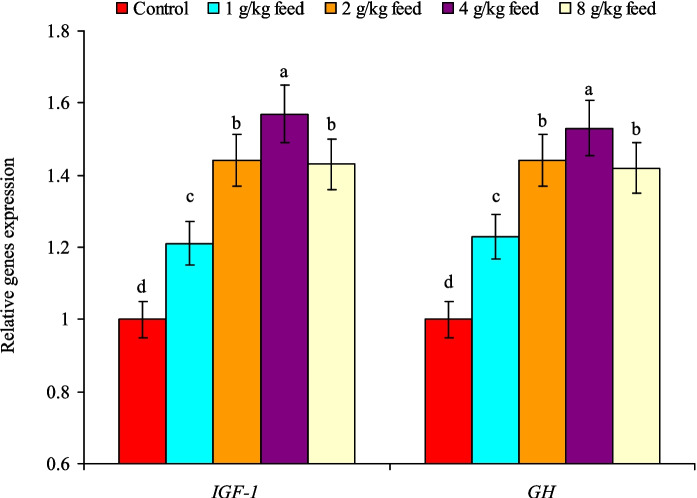
Relative change expression levels of *IGF-1* and *GH* genes in anterior kidney tissues of Nile tilapia (*O. niloticus*) fed on a commercial acidifiers blend (CAB) for 60 days. Bars of each gene expression having different letters are significantly different at *P* < 0.05

It is noted that feeding Nile tilapia with CAB diets showed significant (*P* < 0.05) upregulation of growth-linked genes (*IGF-1* and* GH*), and their highest expression was detected at a treatment of 4.0 g CAB/kg feed (Fig. 3). The expression of both genes in this treatment was higher than those of the control group by 1.57 times for the *IGF-1* gene and 1.53 times for the* GH* gene. Conversely, fish fed on the control diet had the minimum expression of growth-related genes (Fig. 3).

### Hemato-biochemical indices

When comparing with the control group, the dietary CAB quadratically increased WBCs counts with no changes among CAB-treated groups; meanwhile, RBCs and Hb values were maximized at treatments of 2–8 g CAB/kg feed. Ht levels showed marked increases up to 4.0 g/kg feed, while 8.0 g/kg feed exhibited lower values as 1.0–2.0 g/kg feed, but it was over the control one (Table 4). MCV, MCH, and MCHC values remained unaffected (*P* > 0.05) due to dietary CAB, and their ranges are 96.6–100.6 fL, 54.0–56.7 pg, and 56.4–57.1 g/dL, respectively (Table 4). Serum analyses revealed quadratic (*P* < 0.05) improvements in TP, ALB, and GLOB levels with increasing CAB levels up to 4.0 and 8.0 g/kg feed, and their lowest values were found in the control treatment (Table 5). Glucose, AST, and ALT values exhibited no noted (*P* > 0.05) response to dietary CAB concentrations, and their ranges are 141.6–145.5 mg/dL, 51.5–52.3 IU/L, and 42.2–42.7 IU/L, respectively (Table 5).

**Table 4:** Changes in hematological indices of Nile tilapia (*O. niloticus*) fed on different levels of a commercial acidifiers blend (CAB) for 60 days

Dietary CAB levels (g/kg feed)	WBCs (× 10^3^ μL)	RBCs (× 10^6^ μL)	Hemoglobin (g/dL)	Hematocrit (%)	MCV (fL)	MCH (pg)	MCHC (g/dL)
0.0 (control)	23.4 ± 0.55 b	1.64 ± 0.032 c	9.3 ± 0.036 b	16.5 ± 0.089 d	100.6 ± 1.688	56.7 ± 0.899	56.4 ± 0.318
1.0	24.5 ± 0.45 ab	1.71 ± 0.0145 bc	9.4 ± 0.015 b	16.8 ± 0.046 cd	98.3 ± 0.572	55.0 ± 0.387	56.0 ± 0.171
2.0	24.7 ± 0.47 ab	1.78 ± 0.012 ab	9.7 ± 0.075 ab	17.2 ± 0.092 b	96.6 ± 0.141	54.5 ± 0.557	56.4 ± 0.139
4.0	25.6 ± 0.33 a	1.82 ± 0.009 a	10.1 ± 0.156 a	17.7 ± 0.029 a	97.3 ± 0.542	55.5 ± 0.884	57.1 ± 0.799
8.0	24.7 ± 0.41 ab	1.76 ± 0.022 ab	9.5 ± 0.064 ab	17.1 ± 0.089 bc	97.2 ± 1.191	54.0 ± 0.263	55.6 ± 0.457
*P* value							
Linear	0.921	0.078	0.487	0.105	0.260	0.229	0.591
Quadratic	0.013	0.001	0.009	< 0.001	0.213	0.403	0.770

Mean values followed by different letters in the same column are significantly different at *P* < 0.05

**Table 5:** Changes in the levels of blood total protein (TP), albumin (ALB), globulin (GLOB), glucose, AST, and ALT of Nile tilapia (*O. niloticus*) fed on a commercial acidifiers blend (CAB) for 60 days

Dietary CAB levels (g/kg feed)	TP (mg/dL)	ALB (mg/dL)	GLOB (mg/dL)	Glucose (mg/dL)	AST (IU/L)	ALT (IU/L)
0.0 (control)	19.4 ± 0.59 c	12.6 ± 0.26 c	6.8 ± 0.34 b	142.5 ± 1.39	51.8 ± 0.81	42.5 ± 0.54
1.0	21.9 ± 0.35 b	14.9 ± 0.41 b	7.0 ± 0.58 b	145.5 ± 2.93	51.5 ± 0.49	42.3 ± 0.52
2.0	23.1 ± 0.53 b	16.1 ± 1.17 ab	7.0 ± 0.72 b	141.6 ± 0.98	52.0 ± 1.21	42.5 ± 0.57
4.0	25.2 ± 0.73 a	17.2 ± 0.35 a	8.0 ± 0.38 a	142.1 ± 2.31	52.3 ± 0.86	42.2 ± 0.86
8.0	23.6 ± 0.72 ab	15.8 ± 0.57 ab	7.8 ± 0.49 ab	141.9 ± 1.99	51.8 ± 0.52	42.7 ± 0.55
*P* value						
Linear	0.058	0.062	0.416	0.476	0.856	0.820
Quadratic	< 0.001	< 0.001	0.032	0.767	0.861	0.882

Mean values followed by different letters in the same column are significantly different at *P* < 0.05

### Antioxidant biomarkers

It is noted that feeding Nile tilapia on CAB diets stimulated significantly (*P* < 0.05) the antioxidant enzymatic capacity along with reduced MDA contents (*P* < 0.05; Table 6). The highest SOD, CAT, and GPx activity along with lowest MDA levels were detected in treatments of 4.0 and 8.0 g CAB/kg feed with no significant (*P* > 0.05) differences between them. In contrast, fish fed on the control diet exhibited lowest SOD, CAT, and GPx activities along with the highest MDA level (Table 6).

**Table 6:** Changes in hepatic antioxidant activity and blood immune biomarkers of Nile tilapia (*O. niloticus*) fed on a commercial acidifiers blend (CAB) for 60 days

Dietary CAB levels (g/kg feed)	Hepatic MDA (nmol/mg protein)	Hepatic SOD (mg/mg protein)	Hepatic CAT (mg/mg protein)	Hepatic GPx (mg/mg protein)	LYZ (U/mL)	RBA (µg/mL)	Total Ig (mg/mL)
0.0 (control)	32.7 ± 1.13 a	16.7 ± 0.23 d	10.9 ± 0.15 d	15.7 ± 0.62 d	4.2 ± 0.18 d	0.381 ± 0.021 d	2.51 ± 0.087 c
1.0	29.2 ± 0.95 b	19.1 ± 0.67 c	14.8 ± 0.27 c	17.8 ± 0.23 c	5.1 ± 0.09 c	0.448 ± 0.024 c	3.43 ± 0.121 b
2.0	25.4 ± 0.57 c	22.1 ± 0.47 b	18.6 ± 0.48 b	20.4 ± 1.09 b	6.9 ± 0.13 b	0.519 ± 0.014 b	4.17 ± 0.127 b
4.0	20.2 ± 1.04 d	24.3 ± 0.59 a	22.9 ± 0.73 a	24.3 ± 0.53 a	8.1 ± 0.11 a	0.693 ± 0.053 a	5.13 ± 0.116 a
8.0	19.8 ± 0.79 d	23.2 ± 0.82 ab	21.2 ± 0.99 ab	23.4 ± 0.57 ab	7.9 ± 0.18 a	0.617 ± 0.026 a	5.09 ± 0.217 a
*P* value							
Linear	< 0.001	0.001	0.001	< 0.001	< 0.001	0.003	< 0.001
Quadratic	< 0.001	< 0.001	< 0.001	< 0.001	< 0.001	< 0.001	< 0.001

Mean values followed by different letters in the same column are significantly different at *P* < 0.05

*MDA*, malondialdehyde; *SOD*, superoxide dismutase; *CAT*, catalase; *GPx*, glutathione peroxidase; *LYZ*, lysozyme; *RBA*, respiratory burst activity; *total Ig*, total immunoglobulin

### Immune indices

Data in Table 6 show that immune indices represented by LYZ, RBA, and total Ig levels were increased (*P* < 0.05) markedly with the increase of CAB levels in fish diets and their highest values were observed at levels of 4.0 and 8.0 g/kg diet, with no marked (*P* > 0.05) differences between them. The minimum values of the abovementioned indices were noted in the control group (Table 6).

### Antioxidant and cytokine expression

**Fig. 4:**
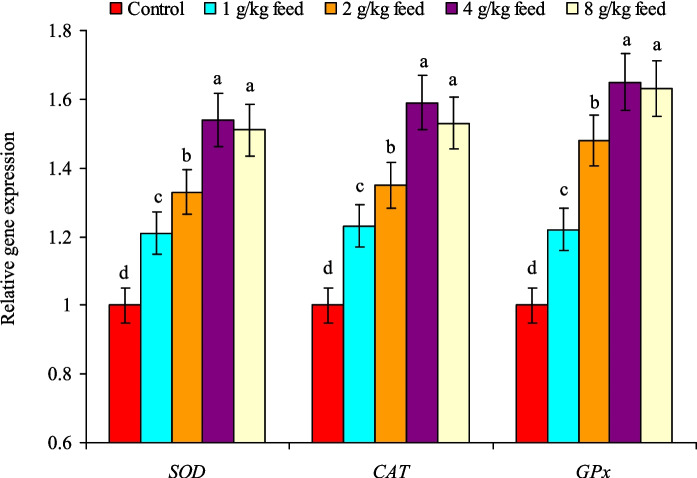
Relative change expression levels of hepatic *SOD*,* CAT*, and *GPx* genes of Nile tilapia (*O. niloticus*) fed on a commercial acidifiers blend (CAB) for 60 days. Bars of each gene expression having different letters are significantly different at *P* < 0.05

**Fig. 5:**
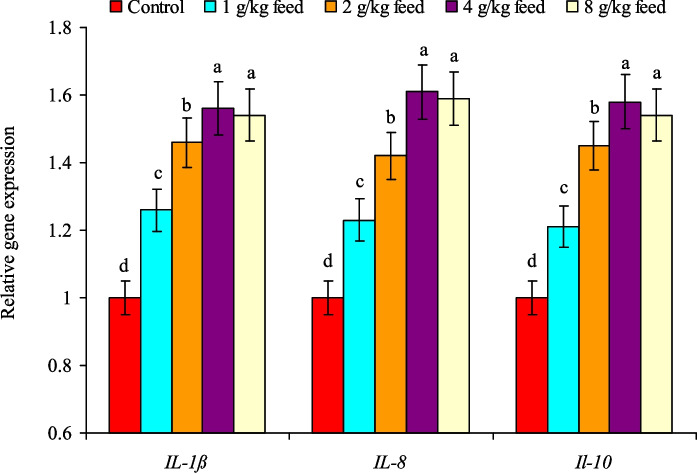
Relative change expression of *IL-1ß*,* IL-8*, and *IL-10* genes in anterior kidney tissues of Nile tilapia (*O. niloticus*) fed on a commercial acidifiers blend (CAB) for 60 days. Bars of each gene expression having different letters are significantly different at *P* < 0.05

Nile tilapia received dietary CAB levels exhibited marked (*P* < 0.05) upregulation of hepatic *SOD*, *CAT*, and* GPx* genes, and their highest expressions were found at treatments of 4.0–8.0 g CAB/kg feed (Fig. 4). The expression of those genes in both treatments was higher than those of the control group by 1.54 and 1.51 times for the *SOD* gene, 1.59 and 1.53 times for the *CAT* gene, and 1.65 and 1.63 times for the* GPx* gene, respectively (Fig. 4). Similarly, the *IL-1β*, *IL-8*, and* IL-10* genes expression was gradually (*P* < 0.05) upregulated with the increase in CAB levels in fish diets. Their highest expression was noted at treatments of 4.0–8.0 g CAB/kg feed with no noted differences between them (Fig. 5). The expression of those genes in both treatments was higher than those of the control group by 1.56 and 1.54 times for the *IL-1β* gene, 1.61 and 1.59 times for the *IL-8* gene, and 1.58 and 1.54 times for the *IL-10* gene, respectively (Fig. 5).

### Antimicrobial efficacy and microbial challenge

**Fig. 6:**
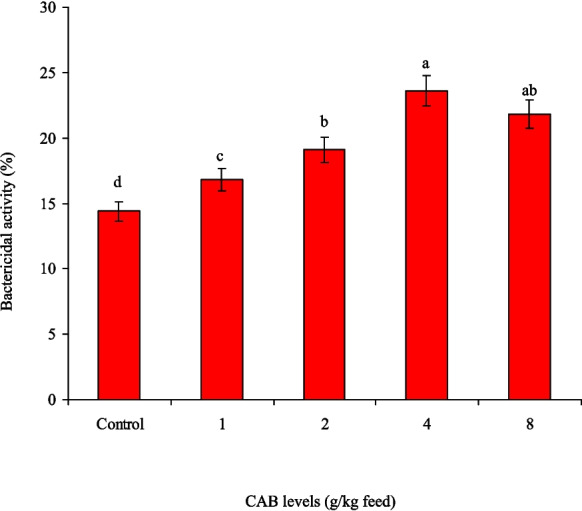
Serum bactericidal activity of Nile tilapia (*O. niloticus*) fed on a commercial acidifiers blend (CAB) for 60 days against *A. sobria*. Bars having different letters are significantly different at *P* < 0.05

**Fig. 7:**
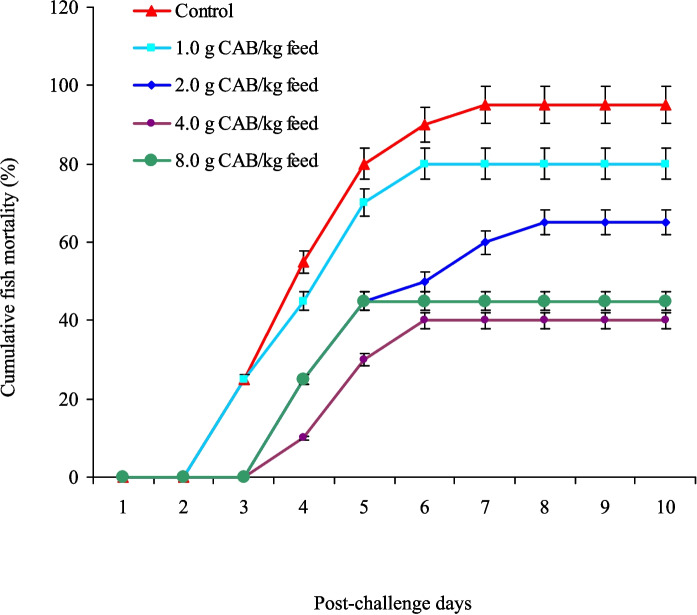
The cumulative mortality (%) of Nile tilapia (*O. niloticus*) fed on a commercial acidifiers blend (CAB) for 60 days and post-challenged with *A. sobria* infection for further ten days

**Fig. 8:**
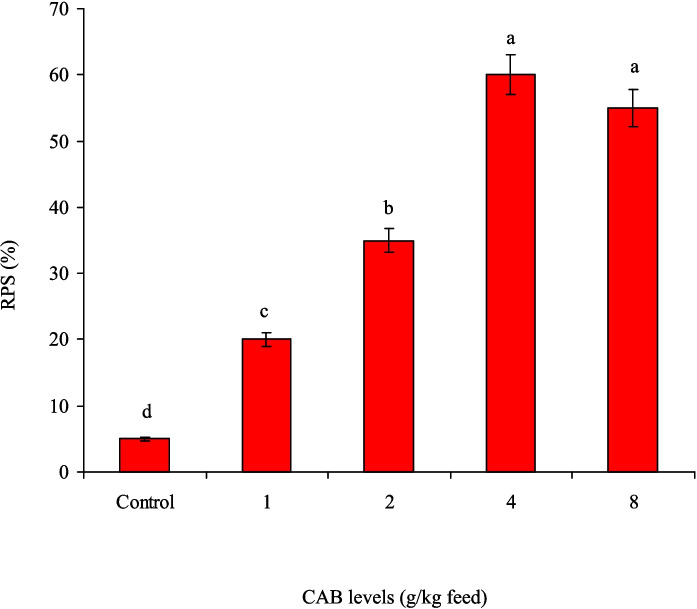
The relative percent survival (RPS) of Nile tilapia (*O. niloticus*) fed on a commercial acidifiers blend (CAB) for 60 days and post-challenged with *A. sobria* infection for further 10 days. Bars having different letters are significantly different at *P* < 0.05 (Tukey test)

After the feeding trial (60 days), Nile tilapia that received 4.0 and 8.0 g CAB/kg diet showed the highest in vitro bactericidal activity against *A. sobria*; meanwhile, the control group had the lowest bactericidal activity (Fig. 6). Following an *A. sobria* challenge, Nile tilapia that received 4.0 and 8.0 g CAB/kg diet had the minimum mortality rates (40% and 45%, respectively), with no noted (*P* > 0.05) differences between them (Fig. 7). In contrast, the control group had the highest mortality rate (90%). It is also noted that feeding Nile tilapia on CAB diets for 60 days and post-challenged with *A. sobria* infection enhanced their RPS, especially at 4.0 (60%) and 8.0 g CAB/kg feed (55%) with no noted differences between them (Fig. 8). Clinically infected fish exhibited symptoms such as fin damage, scale loss, hemorrhaging, and organ congestion.

## Discussion

The ongoing investigation has shown that adding 4.0 g CAB/kg feed to high SBM-based diets significantly improved the growth results in Nile tilapia fingerlings. This highlights the positive effect of dietary CAB in reducing the adverse effects associated with high SBM inclusion in fish diets. Previous research on dietary acidifiers emphasizes their ability to mitigate SBM-induced enteritis, enhance digestion, and maintain gut health and morphology (Lin and Cheng 2017; Liu et al. 2019; Torrecillas et al. 2024; Abang Zamhari et al. 2025; Ruenkoed et al. 2025). In a related study, Ruenkoed et al. (2025) fed Nile tilapia (7.12 ± 0.47 g) with diets containing acidifier blends (ACD) at levels of 0.0 (control), 1.0, and 2.0 g ACD per kg of feed over a period of 56 days. Their findings indicated that increasing the dietary levels significantly enhanced growth performance alongside elevated secretion of proteases, amylase, and lipase. Abang Zamhari et al. (2025) reported that the addition of 3% citric acid and 1% butyric acid to SBM-based diets for juvenile hybrid groupers (*Epinephelus fuscoguttatus* × *E. lanceolatus*) improved nutrient digestibility. Furthermore, dietary acidifiers can enhance phosphorus utilization, leading to reduced phosphorus waste and fostering a better environment that may enhance the antioxidant capacity of fish (Koh et al. 2016; Ng and Koh 2017; Zhang et al. 2022; Hoseini et al. 2025).

The observed increase in growth of Nile tilapia, *O. niloticus*, when fed on diets supplemented with CAB, is linked to heightened activity of digestive enzymes in the fish intestine. This phenomenon may be attributed to the acidifying characteristics of CAB, which significantly contribute to the reduction of gut pH, the stimulation of enzymes secretion, and the modulation of beneficial gut microbiota in fish, such as lactic acid bacteria that produce digestive enzymes in Nile tilapia (Su et al. 2014; Ng and Koh 2017; Sotoudeh et al. 2020; Assan et al. 2022). Additionally, the improved growth observed in CAB-fed fish correlates with significant enhancements in intestinal histomorphometry and improved nutrient digestion and absorption (Ng and Koh 2017; Busti et al. 2020; Sotoudeh et al. 2020; Ruenkoed et al. 2025). These results are consistent with earlier studies that have shown the beneficial effects of dietary acidifiers on the secretion of digestive enzymes and overall fish performance (Huang et al. 2022; Monier et al. 2022; El Sayed et al. 2025; Ruenkoed et al. 2025).

Similar experiments utilized a mixture of acidifiers as feed supplements. In this regard, Reda et al. (2016) indicated that Nile tilapia fingerlings were given feeds containing an acidifier mixture (formic acid and propionic acid/salt) for a duration of 60 days, which demonstrated improved fish performance at a treatment level of 2 g/kg feed compared to other treatments. Abdel-Tawwab et al. (2019) noted that feeding freshwater fishes, which were polycultured in earthen ponds with an acidifier blend, resulted in enhanced growth and productivity. Huang et al. (2022) administered diets containing an acidifier blend (AB; phosphoric, lactic, and citric acids) to juvenile Japanese seabass (*Lateolabrax japonicus*) at varying levels of 0, 1, 2, 3, 4, and 5 g/kg feed for 28 days, revealing that 2 and 4 g AB/kg feed improved growth indices. A study conducted by Zhang et al. (2022) investigated the effects of a commercial acidifiers mixture at concentrations ranging from 0 to 5.0 g/kg feed on American eel juveniles. Their results indicated that 4.0 g/kg feed produced the most advantageous outcomes, including enhanced growth performance, decreased serum lipid levels, and improved liver and immune functions. Zhao et al. (2024) provided largemouth bass (*Micropterus salmoides*) juveniles with high-carbohydrate diets supplemented with sodium acetate (SA), sodium butyrate (SB), and their mixture (2 g/kg feed) for 8 weeks. They found that both acidifiers negatively affected gut health and mitigated intestinal damage by lowering endoplasmic reticulum stress. Hoseini et al. (2025) reported that common carp (*Cyprinus carpio*) juveniles fed on varying levels of a mixture of citric and lactic acids, along with sodium sorbate (1:1:1), exhibited improved gut health, as well as enhanced humoral and mucosal immunity, in addition to overall growth efficiency, particularly at a concentration of 5 g/kg feed.

Notably, administering 8 g CAB/kg of diet to Nile tilapia negatively impacted their growth and digestive enzymes performances. That was possibly attributed to decreased feed consumption (Sardar et al. 2020). Excessive CAB levels may also lower digestive tract pH, potentially damaging the gastrointestinal mucosa (Koh et al. 2016; Ng and Koh 2017; Sardar et al. 2020). Similar negative effects have been observed in other studies, where high levels of acidifier supplementation impaired growth and digestive enzymes activity in various fish species, including Japanese sea bass and juvenile American eel (Huang et al. 2022; Zhang et al. 2022).

The hemato-biochemical assay serves as a crucial tool for evaluating the response of fish to feed utilization. This research indicated that the hematological indices (WBCs, RBCs, Hb, and Ht) in Nile tilapia that were given CAB-enriched feeds showed marked improvements, which may result from the minerals released from feed ingredients, aided by dietary acidifiers (Zhang et al. 2022; El Sayed et al. 2025; Hoseini et al. 2025). Additionally, elevated serum levels of TP, ALB, and GLOB were noted in fish consuming CAB diets, particularly at 4.0 g/kg feed. These outcomes could be linked to better feed intake and enhanced protein synthesis, potentially involving immune-related compounds indicating an improvement in immune functions (Wiegertjes et al. 1996). On the other hand, no significant changes were observed in glucose, AST, and ALT levels among the various CAB-treated fish. These findings suggest that dietary CAB does not adversely affect fish health, indicating its safety for Nile tilapia. In this context, Monier et al. (2022) reported no alterations in glucose, AST, and ALT levels in Nile tilapia fed with nanosized sodium butyrate over an 8-week period. Furthermore, El Sayed et al. (2025) noted significant reductions in glucose, AST, and ALT in red tilapia that were given a blend of acidifiers for 8 weeks.

The antioxidant enzymes (SOD, CAT, and GPx) play a crucial role in maintaining redox balance and regulating the antioxidant process (Livingstone 2001; Abdel-Tawwab and Wafeek 2017; Hoseinifar et al. 2021). In this study, the inclusion of dietary CAB, particularly at a level of 4.0 g/kg feed, improved antioxidant indices and reduced lipid peroxidation (MDA concentration) in Nile tilapia fingerlings. These findings are linked to the upregulation of the expression of *SOD*, *CAT*, and* GPx* genes. The stimulated antioxidant capacity in fish may be attributed to the feed preservation benefits of acidifiers, as dietary acidifiers like CAB can help maintain feed quality, prevent spoilage, and minimize the production of harmful substances, while also enhancing the fish’s internal defense systems against oxidative damage (Koh et al. 2016; Ng and Koh 2017). Similar enhancements in antioxidant indices alongside reductions in lipid peroxidation were noted in fish that were fed dietary acidifiers (Chen et al. 2018; Hoseini et al. 2022, 2023, 2025). The antioxidant-regulating effects of dietary acidifiers may be associated with the intestinal microbiota of fish, as earlier research indicates that beneficial bacteria can boost antioxidant capacity (Hoseinifar et al. 2021).

The cellular and innate immune functions serve as crucial defense mechanisms in fish (Secombes and Fletcher 1992; Magnadóttir 2006; Saurabh and Sahoo 2008). This research indicates that dietary CAB has improved immune functions in Nile tilapia, as shown by the rise in LYZ, RBA, and total Ig values, along with the upregulation of mRNA expression for immune-related genes. These enhancements may be linked to the support of beneficial intestinal microbiome, which reduces harmful bacterial populations and regulates immune-related genes in fish (Koh et al. 2016; Ng and Koh 2017). Furthermore, the lower pH levels in fish guts resulting from acidifiers consumption inhibit the growth of pathogenic microorganisms; thereby, enhancing the fish immune functions, as demonstrated in a study involving rainbow trout, *Oncorhynchus mykiss* (Yilmaz et al. 2018). Notably, Zhang et al. (2022) suggested that dietary CAs improved the immune function in juvenile American eel, with dietary CAs leading to increased levels of LZM, C3, and IgM in fish.

In fish, cytokines such as *IL-1β*, *IL-8*, and* IL-10* play a crucial role in modulating the immune response (Yin et al. 1997; Llopiz et al. 2017; Oliver et al. 2023). The present study revealed that dietary CAB increased the expression levels of *IL-1β*, *IL-8*, and* IL-10* in Nile tilapia, indicating a strengthened immune response. Reda et al. (2016) identified a positive correlation between dietary acidifiers and cytokine production in Nile tilapia. For example, a mixture of citric acid, lactic acid, and potassium sorbate enhanced the expression of *tumor necrosis factor-alpha* (*TNF-α*), *IL-1β*, *LYZ*, and* HSP70* genes in common carp (Hoseini et al. 2025). Likewise, dietary malic acid and butyrate have been shown to increase the expressions of *TNF-α*, *LYZ*, and* IL-1β* in rainbow trout (Taheri Mirghaed et al. 2019; Yousefi et al. 2023). Furthermore, the increased expression of *LYZ*, *IL-1β*, and* TNF-α* genes in the intestines of fish following dietary supplementation with organic acids/salts has been linked to improved disease resistance, as demonstrated in studies involving rainbow trout (Taheri Mirghaed et al. 2019) and crucian carp (Li et al. 2022).

The use of acidifiers as dietary supplements is increasingly recognized in aquaculture as a preventive strategy against pathogens. This research demonstrated that dietary CAB, particularly at concentrations of 4.0 and 8.0 g/kg feed, significantly inhibited the pathogenic *A. sobria* both in vitro and in vivo. The antimicrobial properties of CAB against *A. sobria* can be attributed to the enhancement of immune functions, as indicated by LYZ activity, RB activity, and total Ig levels. The antibacterial properties of dietary acidifiers derive from their capacity to penetrate the cell walls of gram-negative microorganisms, disrupting their internal pH balance and inducing apoptosis (Defoirdt et al. 2009). Furthermore, dietary acidifiers can disrupt bacterial DNA synthesis and nutrient absorption processes, thereby hindering their growth and reproduction or directly killing the bacteria (Silliker and Silliker 1980). In this context, Reda et al. (2016) noted that feeding Nile tilapia with an acidifiers mixture suppressed the growth of pathogenic *A. sobria *in vitro. Similarly, da Silva et al. (2013) found that acidifiers such as sodium acetate and sodium butyrate inhibited the growth of pathogenic *Vibrio* species in vitro. Fujiki et al. (1994) reported that feeding common carp, *C. carpio*, on diets with sodium alginate improved its resistance to *Edwardsiella tarda* infection. Moreover, supplementing the diets of Nile tilapia with acidifiers has been shown to enhance their survival rates when faced with *Streptococcus agalactiae* challenges, underscoring the beneficial effects of these supplements in aquaculture (Koh et al. 2016; Ng and Koh 2017).

## Conclusion

The present investigation revealed that the inclusion of 4.0 g CAB/kg feed is the optimum CAB level, which enhanced the growth, antioxidant, and immune performances as well as the fish resistance against the *A. sobria* infection. Feeding Nile tilapia fingerlings on 4.0 g CAB/kg feed upregulated the expression of growth-, antioxidant-, and immune-related genes. Further applications of CAB-enriched diets in field trials are needed, and further studies should be done to explore the CAB effects on gut microbiota that may play a crucial role in enhancing fish performance.

## Data Availability

No datasets were generated or analysed during the current study.
